# Neurons Are Not All the Same: Diversity in Neuronal Populations and Their Intrinsic Responses to Spinal Cord Injury

**DOI:** 10.1080/17590914.2024.2440299

**Published:** 2025-01-16

**Authors:** Justin R. Siebert, Kiersten Kennedy, Donna J. Osterhout

**Affiliations:** a Physician Assistant Studies Program, Department of Health Care and Administration, Slippery Rock University of Pennsylvania, Slippery Rock, PA, USA; b Norton College of Medicine, SUNY Upstate Medical University, Syracuse, NY, USA; c Department of Cell & Developmental Biology, SUNY Upstate Medical University, Syracuse, NY, USA

**Keywords:** Corticospinal, dorsal root ganglion, propriospinal, rubrospinal, vestibulospinal

## Abstract

Functional recovery following spinal cord injury will require the regeneration and repair of damaged neuronal pathways. It is well known that the tissue response to injury involves inflammation and the formation of a glial scar at the lesion site, which significantly impairs the capacity for neuronal regeneration and functional recovery. There are initial attempts by both supraspinal and intraspinal neurons to regenerate damaged axons, often influenced by the neighboring tissue pathology. Many experimental therapeutic strategies are targeted to further stimulate the initial axonal regrowth, with little consideration for the diversity of the affected neuronal populations. Notably, recent studies reveal that the neuronal response to injury is variable, based on multiple factors, including the location of the injury with respect to the neuronal cell bodies and the affected neuronal populations. New insights into regenerative mechanisms have shown that neurons are not homogenous but instead exhibit a wide array of diversity in their gene expression, physiology, and intrinsic responses to injury. Understanding this diverse intrinsic response is crucial, as complete functional recovery requires the successful coordinated regeneration and reorganization of various neuron pathways.

## SUMMARY STATEMENT

Neurons in the brain and spinal cord are a diverse group of cells with many populations and subtypes. To facilitate recovery of function post-injury, understanding the unique individual responses of these neurons to injury is going to be critical.

## Introduction

After a spinal cord injury, the therapeutic goal is to regenerate the injured neural pathways, restoring not only neuronal connections but also essential sensory and autonomic circuits to fully restore functionality. Numerous studies using experimental models of spinal injury have identified several approaches that can promote nerve repair. These approaches include cellular and molecular changes in the local microenvironment and the use of factors that enhance the survival and sprouting of individual neuronal populations in the adult central nervous system (CNS) (reviewed by Mahar & Cavalli, 2018; Bradbury & Burnside, 2019; Fawcett, 2020; Tran et al., 2022). However, subtle molecular differences between neuronal pathways have been identified in earlier studies. New techniques, including genomics and proteomic analyses, combined with our previous knowledge, provide a clearer overview of the regenerative capacity of individual neuronal pathways and their variability in response to injury. Acknowledging this intrinsic variability in neuronal pathways and populations is pivotal for developing successful strategies to restore optimal motor function.

### Anatomical Organization of Neuronal Populations

The CNS includes both the brain and spinal cord, comprising a multitude of neuronal populations. Neurons can be largely grouped into either the afferent or efferent classes, which give rise to the ascending (sensory) or descending (motor) pathways. The spinal cord links the brain to the body, transmitting afferent and efferent signals. However, with its plethora of interneuron populations, it is important to note that the spinal cord transcends its role as a mere biological conduit subservient to the brain. It can autonomously process both sensory and motor information and coordinate a spectrum of walking behaviors. This is observed in spinalized animal models, where the animals maneuver at different speeds and directions on a treadmill with appropriate weight support (Martinez et al., 2013; Côté et al., 2018; Merlet et al., 2021).

**Figure 1.**
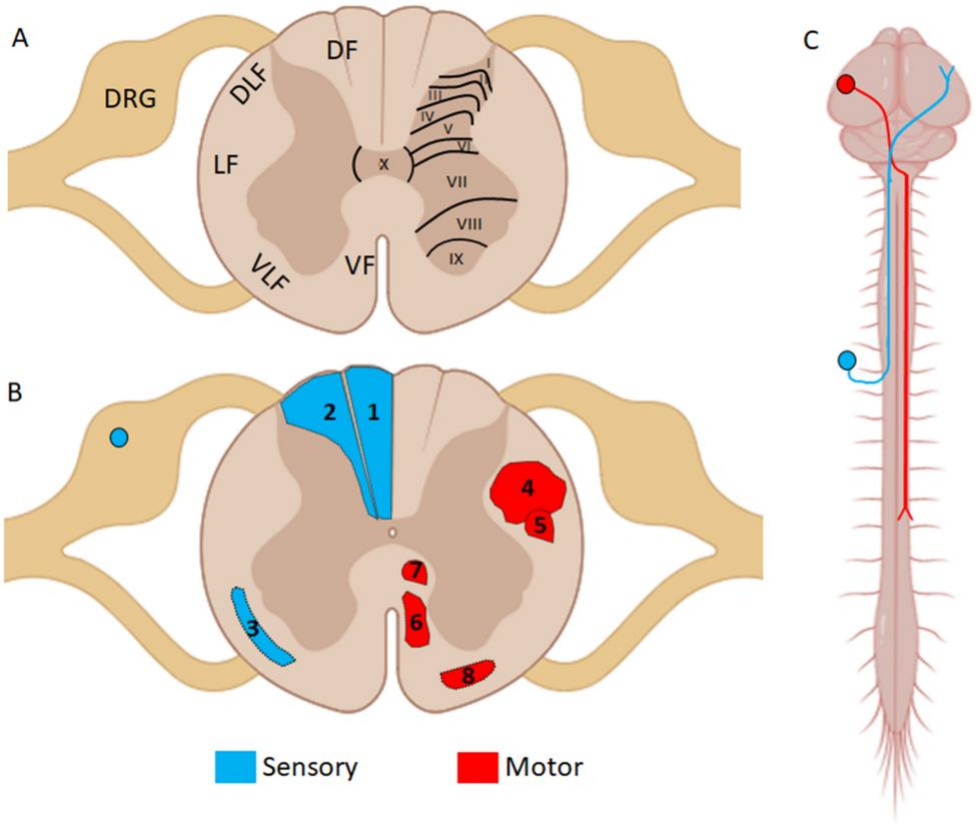
Neuroanatomical organization of the spinal cord. (a) Schematic illustrating the location of important sensory and motor pathways of the human spinal cord. The white matter is divided into structures known as funiculi. DRG - dorsal root ganglion, LF- lateral funiculus, DLF- dorsolateral funiculus, DF-dorsal funiculus, VF- ventral funiculus. The gray matter is divided into layers known as lamina. There are 10 lamina of the spinal gray matter which are represented in roman numerals. (b) Topographic map of the motor and sensory pathways found in the human spinal cord. Descending motor pathways are represented in red, and include 4-Lateral CST, 5-RST, 8-VST, 6-Anterior CST, 7-ReST. Ascending sensory pathways are represented in blue and include the 1-Cuneate funiculus, 2-Facile funiculus, and 3-plAnterolateral system or Spinothalamic tract. The DRG neurons are located outside of the spinal cord; represented by the blue circle. *Created with BioRender.com.*

Like the brain, the spinal cord contains both white and gray matter, with the gray matter lying centrally, surrounded by the white matter. Resembling the shape of a butterfly or the letter H, the gray matter is divided into various zones known as laminae, while the white matter is divided into regions known as funiculi (Blumenfeld, 2010; Haines, 2012; Figure 1a). In humans, the afferent and efferent pathways are highly organized and located in distinct areas of the central nervous system, resulting in differential vulnerabilities to lesions or trauma in the CNS. Importantly, to achieve complete recovery of function, repair of both the afferent and efferent pathways is necessary.

Efferent neuronal populations, including the corticospinal tract (CST) neurons, rubrospinal tract (RST) neurons, vestibulospinal tract (VST) neurons, and reticulospinal tract (ReST) neurons, originate from the brain and brainstem and project their axons down through the spinal cord; thus, they are categorized as supraspinal neurons (SSNs). In humans, the CST is the most significant motor pathway, crucial for voluntary movement. The CST arises primarily from the frontoparietal cortices, encompassing the primary motor cortex, secondary motor area, and somatosensory cortex (Natali et al., 2023). Axons from CST neurons project caudally through the brainstem, where they decussate, and then continue caudally down the lateral aspect of the spinal cord to their peripheral targets (Blumenfeld, 2010; Mtui et al., 2016; Figure 1b). Rubrospinal neurons form a relatively minor motor pathway that innervates and influences the flexor muscle groups, mainly in the upper regions of the spinal cord. Arising from the red nucleus in the midbrain region, this pathway projects through the ventral tegmental area of the brain and then descends into the spinal cord, running adjacent to the CST (Blumenfeld, 2010; Mtui et al., 2016; Figure 1b). Vestibulospinal neurons arise from two different brain regions, with the lateral vestibulospinal neurons originating from the lateral vestibular nucleus in the pons and the medial vestibulospinal neurons originating from the medial vestibular nucleus in the rostral medulla; both populations of vestibulospinal neurons project down into the spinal cord (Blumenfeld, 2010). These two pools of VST neurons give rise to the medial and lateral vestibulospinal tracts, which are important not only in coordinating postural extensor activity and maintaining the center of the body’s gravity between the feet but also in coordinating the position of the head with the body in space (Watson & Harvey, 2009; Blumenfeld, 2010; Mtui et al., 2016). Reticulospinal neurons, which arise in the pontine reticular formation and project down into the spinal cord, are involved in postural and locomotor control (Mtui et al., 2016). Descending efferent tracts are primarily located in either the lateral funiculus (CST and RST) or ventral funiculus (VST and ReST; Blumenfeld, 2010; Haines, 2012).

Afferent or sensory neuronal populations include the dorsal root ganglion (DRG) cells, which are located just outside the spinal cord in an adjacent structure known as the dorsal root ganglion. DRG neurons have an axon that bifurcates into two main branches: a long peripheral process that projects into the peripheral nerve and a central process, called the dorsal root, which projects into the spinal cord through the dorsal root entry zone into the dorsal horn (Laminae I-IV). Sensory information from spinal level T_6_ and caudal travels within the gracile fasciculus, while afferent information from spinal level T_6_ and rostral travels within the cuneate fasciculus (Blumenfeld, 2010; Haines, 2012; Mtui et al., 2016). Both sensory tracts are located in the dorsal funiculus of the spinal cord and are known as the dorsal columns (Blumenfeld, 2010; Haines, 2012). In addition to the dorsal columns, a more primitive pain/temperature/crude touch pathway, the anterolateral tract, ascends within the ventrolateral funiculus (Figure 1b). The anterolateral tract and several other afferent pathways relay different types of sensory information and arise from neurons within the dorsal horn (Laminae I–VI) or intermediate gray matter (Laminae VII, VIII). These pathways travel through various regions of the white matter, projecting to different supraspinal nuclei, ultimately relaying sensory information to their respective areas of the brain, which can include the primary sensory cortex and other regions such as the anterior cingulate gyrus (Blumenfeld, 2010; Haines, 2012).

**Figure 2.**
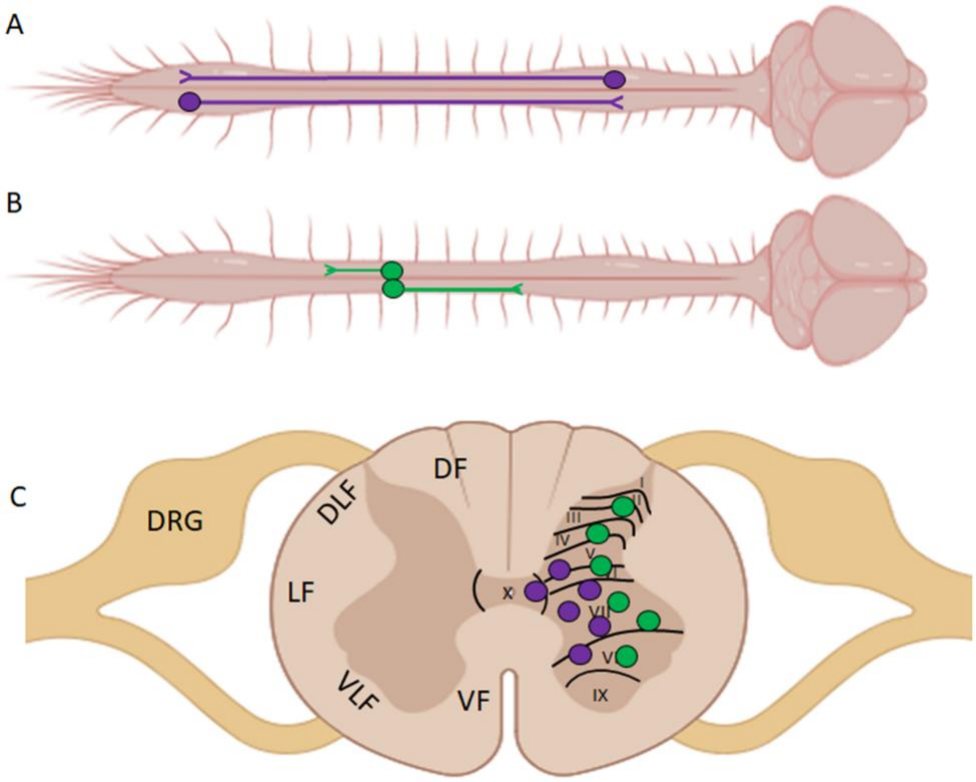
Organization of propriospinal neurons in the spinal cord. (a) LADPT and LDPT neurons have their cell bodies located within the CE and LSE, and project their axons up and down the spinal cord interconnecting the spinal enlargement. (b) TPS neurons have short axons that project rostrally or caudally within the thoracic levels of the spinal cord. (C) General distribution of the propriospinal neurons within the spinal gray matter. *Created with BioRender.com.*

Propriospinal (PS) neurons are a population of interneurons that interconnect different levels of the spinal cord. Unlike SSNs (CST, RST, ReST, VST), which originate in the brain or brainstem and project into the spinal gray matter, PS neurons both originate and terminate within the boundaries of the spinal cord (Chung & Coggeshall, 1983; Figure 2a,b). Anatomical studies have demonstrated that axons of PS origin comprise approximately one-quarter to one-third of the fiber tracts found within the dorsal and dorsolateral funiculus of the rat spinal cord (Chung et al., 1987). Located throughout the various levels of the spinal cord, PS neurons can be classified into two groups: long and short axon PS neurons (Conta & Stelzner, 2004; Conta & Stelzner, 2009). These two groups can then be divided into smaller subgroups such as long ascending PS neurons (LAPT), long descending PS neurons (LDPT), short thoracic PS neurons (TPS), short axon PS neurons, thoracic respiratory PS neurons, and upper cervical respiratory PS neurons. A detailed characterization of the multiple subclasses of PS neurons is described elsewhere (Conta & Stelzner, 2009; Laliberte et al., 2019). LDPT neurons are located within the intermediate gray matter (Laminae VII and VIII), within deeper laminae of the dorsal horn (Laminae V and VI), and surrounding the central canal (Lamina X; see Figure 2c) of the cervical enlargement (CE) (Conta & Stelzner, 2004; Conta & Stelzner, 2009). After extending their axons caudally for many spinal segments, their main axonal projection terminates within the intermediate gray matter of the lumbosacral enlargement (LSE). In a reciprocal fashion, LAPT neurons, originating in the rostral segments of the LSE, project their axons rostrally towards the CE. Once in the CE, the main LAPT projection terminates within the intermediate gray matter, interconnecting both spinal enlargements and allowing for the coordination of upper and lower extremities during locomotion. TPS, as their name suggests, arise from the thoracic spinal cord and can be found in all laminae of the gray matter except Lamina IX (see Figure 2c). These neurons extend their projections from 1 to 5 segments in either the rostral or caudal direction and are believed to control the postural and axial musculature (Conta & Stelzner, 2009; Laliberte et al., 2019).

### Human vs. Murine CNS Anatomy

Many studies examining the intrinsic responses of specific neuronal populations to trauma are conducted in members of the murine family (rats and mice). While these studies have advanced our understanding of neural regeneration, there are anatomical differences between humans and members of the murine family that need to be considered. First, humans utilize the CST as the principal motor pathway, with the RST playing a much more minor role in human locomotion (Watson & Harvey, 2009; Blumenfeld, 2010; Mtui et al., 2016). The CST in humans controls the voluntary movement of contralateral limbs (Lemon, 2019; Natali et al., 2023). However, in murine animals, especially *Rattus norvegicus*, the CST plays a much smaller role in locomotor ability; rats depend more on pathways found in the lateral and ventral white matter (Kjell & Olson, 2016; Morris & Whishaw, 2016). While motor control in rodents is widely known to be heavily reliant on the RST, it is becoming apparent that the CST is integrated with other primary motor pathways and exerts a significant influence on the dexterous ability of forelimb paw movements (Basista & Yoshida, 2020; Serradj et al., 2023).

**Figure 3.**
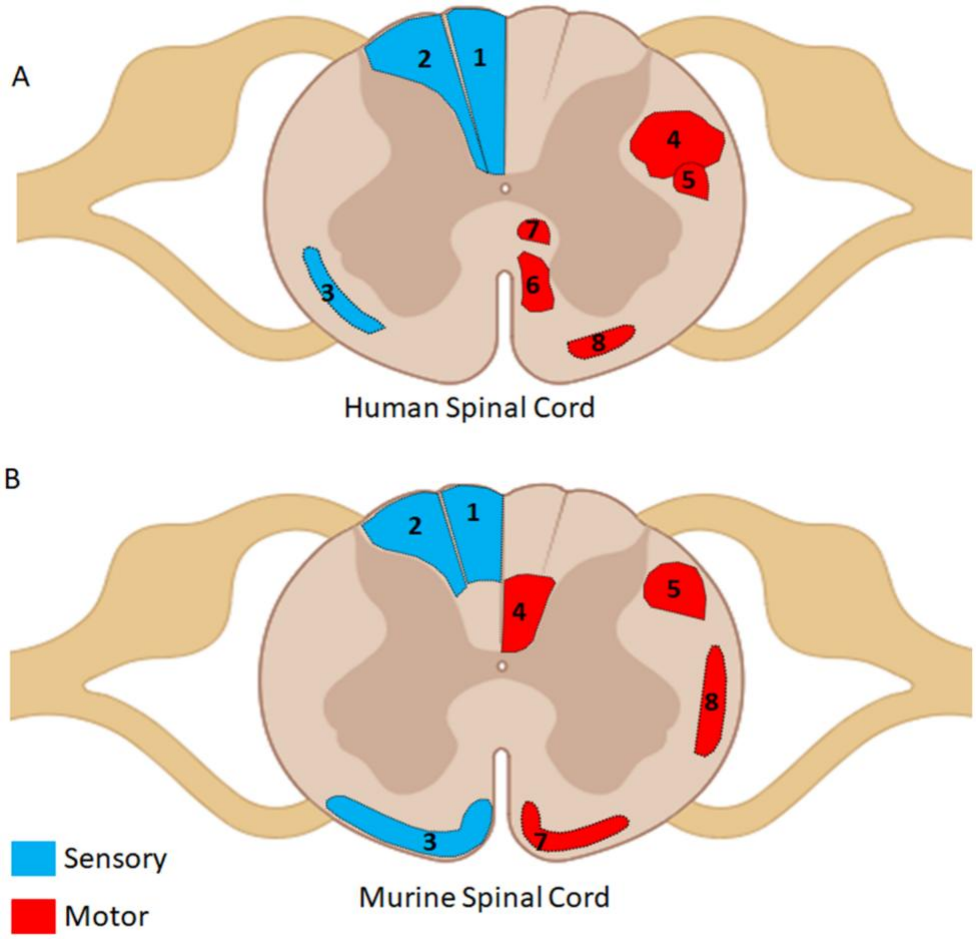
Comparison of human vs. murine spinal cord. (a) Representation of major ascending and descending pathways in the human spinal cord. (b) Representation of major ascending and descending pathways in the murine spinal cord. Note the difference in the locations of the CST between mouse and human. Descending motor pathways are represented in red, and include 4-Lateral CST, 5- RST, 8-VST, 6-Anterior CST, 7-ReST. Ascending sensory pathways are represented in blue and include the 1-Cuneate funiculus, 2-Facile funiculus, and 3-the Anterolateral system or Spinothalamic tract. *Created with BioRender.com.*

Second, the topographic arrangement of these motor pathways differs: in humans, the CST is localized to the lateral funiculus of the spinal cord, while in rats and mice, it is found deep in the dorsal funiculus. However, the RST in both the murine family and humans travels in the lateral funiculus of the spinal cord (Watson & Harvey, 2009; Kjell & Olson, 2016; Morris & Whishaw, 2016; see Figure 3). Furthermore, damage to specific ascending and descending tracts will vary based on the type and location of injury (transection, crush, impaction, etc.) in the spinal cord. Understanding and appreciating the topography of these pathways and their projections is critical for the study and analysis of spinal cord injury (Steward & Willenberg, 2017).

### Pathophysiologic Response of CNS to Injury

Trauma to the CNS triggers dynamic and complex tissue changes that significantly alter neuronal function. The inflammatory response following spinal cord injury (SCI) is robust and begins almost immediately. Activated immune cells, such as microglia and macrophages, infiltrate the lesion site, releasing pro-inflammatory cytokines, chemokines, and other molecules that clear debris and promote tissue repair (Chen & Trapp, 2016; Anjum et al., 2020; Lima et al., 2022; Li et al., 2024). This response peaks within the first week, aiding in debris clearance and tissue repair.

**Figure 4.**
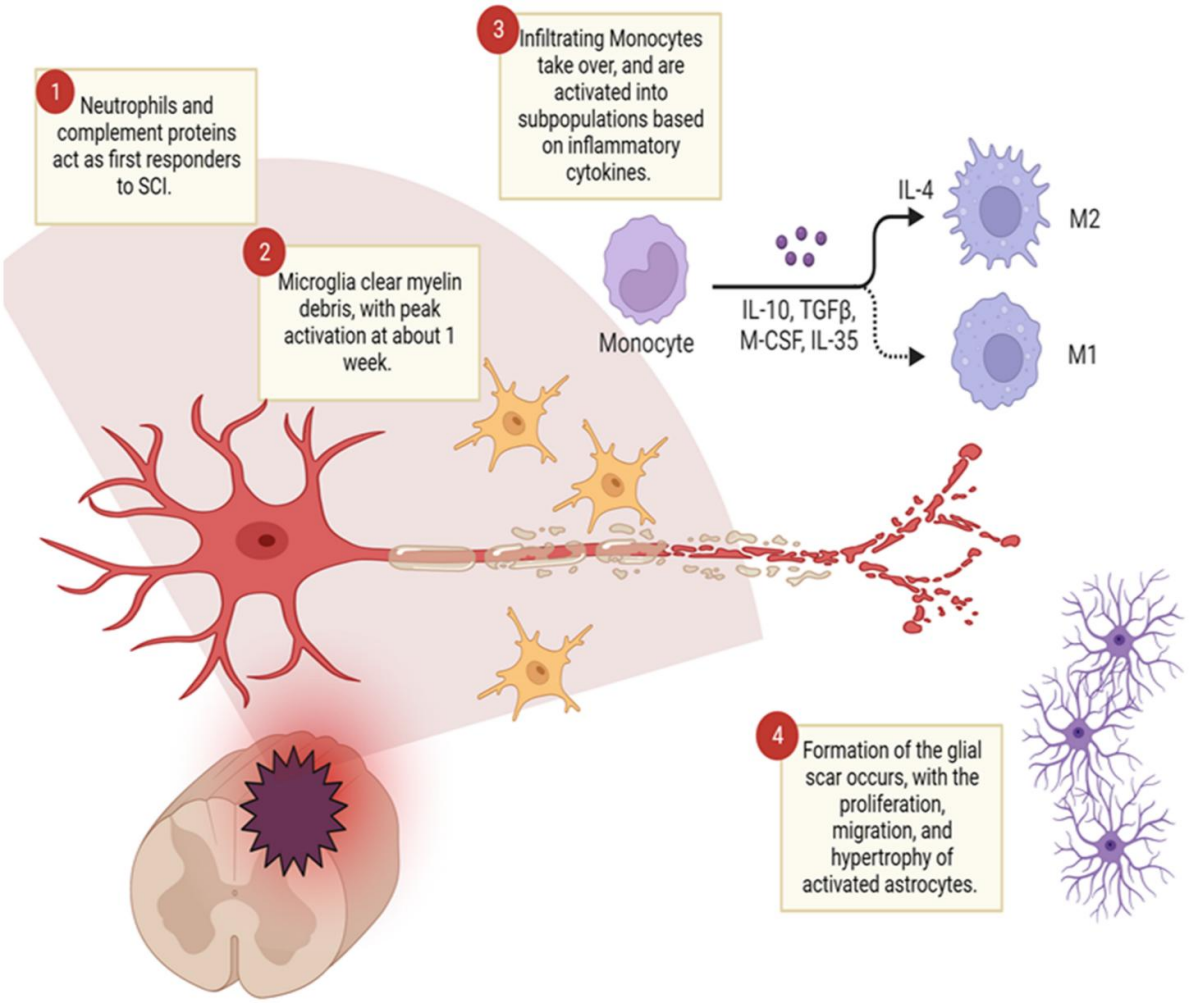
Pathophysiological changes in spinal cord following injury. Schematic illustrating the pathophysiological changes that occur in the spinal cord following injury. *Created with BioRender.com.*

Macrophages, derived from infiltrating monocytes, undergo a progression of phenotypes influenced by their microenvironment (Kopper & Gensel, 2018; An et al., 2021). They are generally categorized into M1 and M2 types. M1 macrophages are cytotoxic to neurons, while M2 macrophages promote regeneration and support axonal outgrowth across inhibitory barriers (Gensel & Zhang, 2015; Hellenbrand et al., 2021; An et al., 2021). In the acute phase of SCI, inflammatory factors like IL-6, TNF-α, IL-1β, and IL-12 drive M1 polarization. Between 1 and 3 days post-injury, increases in IL-4 promote the M2a phenotype, marked by arginase, CD206, and Fizz-1 expression (Gensel & Zhang, 2015; An et al., 2021). Normally, regulatory M2b macrophages would increase and secrete more anti-inflammatory IL-10. However, in SCI, M2b macrophages decline after 3 days, with IL-10 expression significantly reduced by day 6 (Gensel & Zhang, 2015; An et al., 2021). This delayed M2b activation may contribute to chronic inflammation and remodeling seen in SCI for months or years. By 2 to 3 weeks post-injury, the M2c phenotype emerges, characterized by elevated CD206, CD163, and TGF-β expression, although its role is still under investigation (Gensel & Zhang, 2015; An et al., 2021). While the initial inflammatory response is crucial for activating microglia and monocytes, prolonged inflammation can severely impede repair and regeneration (Figure 4).

The inflammatory profile post-injury promotes the formation of the glial scar through astrogliosis and the migration of oligodendrocyte progenitor cells (OPCs) to the lesion core during the acute tissue remodeling phase. Astrocytes play a key role in the injury response, as extensively reviewed (Fawcett & Asher, 1999; Adams & Gallo, 2018; He et al., 2020; Yang et al., 2020; Michinaga & Koyama, 2021; Yu et al., 2021; Zhang et al., 2021; Tran et al., 2022). Immune mediators produced at the lesion initiate changes in astrocyte morphology and function. Astrocytes within the lesion penumbra increase their production of GFAP, hypertrophy, and significantly alter their arrangement and density, contributing to their inhibitory influence on axonal regeneration. However, astrocytes located farther from the lesion epicenter, while upregulating GFAP, do not change their orientation and do not exert the same inhibitory effect on axonal growth (Tran et al., 2022). In addition to activated astrocytes, fibroblasts also contribute to the formation of a fibrotic layer inside the lesion (Tran et al., 2022). This physical barrier is accompanied by the expression of various molecules that inhibit the repair process. Ultimately, this chemical and physical barrier, often referred to as the glial scar, walls off the injured area and protects adjacent healthy tissue.

Numerous studies have identified many cellular and molecular components of the glial scar that impede axonal regeneration in the CNS. A well-known family of inhibitory molecules expressed in and around the lesion is chondroitin sulfate proteoglycans (CSPGs). Structurally, these molecules consist of a protein core with large glycosaminoglycan (GAG) side chains that inhibit axonal growth (Fawcett & Asher, 1999; Silver & Miller, 2004; Siebert et al., 2014; Schwartz & Domowicz, 2018; Hussein et al., 2020; Hu et al., 2022). The protein core of CSPGs interacts with hyaluronan, the primary ECM support protein of the CNS, and with tenascin proteins involved in crosslinking (Bradbury & Burnside, 2019). The growth inhibition is mainly associated with the glycosaminoglycan chains of CSPGs. Enzymatic degradation of these chains with chondroitinase ABC (cABC) eliminates this inhibitory activity and, when applied in vivo, can improve overall axonal regenerative sprouting and growth (Bradbury et al., 2002; Orr & Gensel, 2018; Mukherjee et al., 2020). The assembly of the glial scar progresses through acute, subacute, and chronic phases. Within 24 hours of injury, vascular and tissue damage initiates inflammation, tissue degeneration, and accumulation of cellular debris. This debris accumulates within one week but can persist chronically for over a year, inhibiting axon growth and remyelination (Kopper & Gensel, 2018). The production and deposition of CSPGs at the lesion site also begin within 24 hours and can remain for many months post-injury (Gaudet & Fonken, 2018).

Persistent myelin debris at the injury site stimulates a chronic inflammatory immune response and inhibits axonal growth. As myelin breaks down, it releases several damage-associated proteins (DAMPs), which are intracellular proteins that signal injury and trigger a non-infectious immune response. The presence of DAMPs increases the production of cytokines such as TNFα, perpetuating the inflammatory response. Myelin degradation releases Nogo A, myelin-associated glycoprotein, and oligodendrocyte myelin glycoprotein, all of which bind to axonal receptors and activate internal signaling pathways that cause growth cone collapse, inhibiting axon growth (Bradbury & Burnside, 2019). Thus, the buildup of myelin debris significantly disrupts the regeneration of damaged pathways.

### Neuronal Response to Injury & Factors Influencing Regeneration

The inability of the spinal cord to regenerate and recover following injury is well known, even documented in an ancient Egyptian text, the Edwin Smith Papyrus: “One having a crushed vertebra in his neck; he is unconscious of his two arms and his two legs, and he is speechless. An ailment not to be treated’ (Hughes, 1988). It was not until the studies of Aguayo and colleagues that this belief was first challenged. Their work demonstrated that CNS neurons, particularly those axotomized close to their cell body, could regrow their axons within a peripheral nerve environment (David & Aguayo, 1981; Benfey & Aguayo, 1982). Further studies have revealed that while SSNs lack a strong intrinsic regenerative response following axotomy in the spinal cord, they will attempt to regenerate after injury if the inhibitory post-lesion environmental conditions are modified (e.g., neurotrophic supplementation, neutralization of inhibitory environmental factors) (David & Aguayo, 1981; Benfey & Aguayo, 1982; Fernandes et al., 1999; Mason et al., 2003; Hossain-Ibraham et al., 2006; Zheng & Tuszynski, 2023).

DRG cells have an interesting response to injury, given that they have an axon with branches that project into both the central and peripheral nervous systems. The central dorsal root projection usually does not regenerate within the spinal cord following axotomy; however, if the peripheral process is injured prior to the CNS injury, regenerative growth of dorsal root axons within the spinal cord will occur (see review by Hoffman, 2010). This ‘conditioning lesion’ effect has also been shown in the sympathetic nervous system following axotomy (Shoemaker et al., 2005). It is well documented that axons in the peripheral nervous system can undergo a level of regeneration not seen in the CNS. Many studies have shown that injuring the peripheral branch of the DRG axon primes the DRG for a faster regenerative response (e.g., Li et al., 2009; Hoffman, 2010). A key finding in these studies is the expression and upregulation of a specific set of genes, referred to as regeneration-associated genes (RAGs), in regenerating neurons.

### Regeneration Associated Genes

The homeostatic disruptions caused by an injury trigger a cascade of cellular events that lead to new gene expression. The expression of certain gene products is associated with regenerating neurons in both the PNS and CNS and occurs early after injury. These products are defined as regeneration-associated, as they are typically observed only in neurons sprouting after axotomy. This correlation has been established by examining gene expression in various neuronal populations and measuring axon growth into peripheral nerve grafts alongside gene expression studies (Mason et al., 2002; Zhang et al., 2005).

One of the earliest events following injury is the activation of transcription factors such as *c-Jun*, which regulates the expression of genes involved in axon growth and regeneration. *c-Jun* is upregulated within hours of axotomy and facilitates the upregulation of regeneration-associated genes (RAGs) such as growth-associated protein 43 (*GAP-43*) and neural cell adhesion molecule (*NCAM*) (Raivich & Makwana, 2007; Hanz & Fainzilber, 2006). Additionally, growth factors like nerve growth factor (*NGF*), brain-derived neurotrophic factor (BDNF), and glial cell line-derived neurotrophic factor (*GDNF*) promote the survival and growth of neurons following injury. These factors act via the activation of the RAS/ERK and PI3K/Akt pathways, initiating RAG expression during the regenerative process (Chung et al., 2020; Hausott et al., 2022).

Among the most well-studied RAGs is GAP-43, located in the neuronal growth cone. Animal model studies have demonstrated that overexpression of GAP-43 can enhance axon regeneration and functional recovery after CNS injury (Skene & Willard, 1981; Chung et al., 2020). GAP-43 (growth-associated protein 43) is integral to axonal growth cone function and plays a pivotal role in neuronal growth and regeneration. Predominantly found at axon terminals and growth cones, GAP-43 is crucial for the dynamic processes of axon elongation and navigation. Following neuronal injury, its expression is upregulated, indicating its involvement in the repair and regeneration of damaged neurons. GAP-43 modulates growth cone dynamics by interacting with the cytoskeleton and membrane components, which facilitates growth cone motility. It binds to calmodulin, regulating actin dynamics essential for axon outgrowth, and its phosphorylation by protein kinase C (PKC) further enhances its activity, promoting growth cone advancement (Chung et al., 2020). Other RAGs implicated in axon regeneration include Sox11, ATF3, and STAT3 (Ma & Willis, 2015; He & Jin, 2016).

In addition to promoting axon regeneration, regeneration-associated genes (RAGs) have also been shown to regulate neuronal survival and apoptosis after injury. For example, Bcl-2, a member of the Bcl-2 family of anti-apoptotic proteins, is upregulated in neurons following injury, promoting neuronal survival (Chen et al., 1997; Akhtar et al., 2004; Mi et al., 2004; Park et al., 2008; Wang et al., 2011). While research on RAG discovery and function is ongoing, certain genes have been identified across multiple neuronal populations and are crucial for neuronal survival and/or axonal growth post-axotomy. Table 1 provides a small list of well-known RAGs along with their currently understood functions. It is important to note that the genes listed in Table 1 are upregulated or downregulated following SCI without any experimental manipulation.

**Table 1.** ▪▪▪.

*Gene*	Full name	Functionality	References
Akt	Protein Kinase B	Protein kinase that is involved in the regulation of cell growth and survival. Akt has been shown to play a critical role in promoting the survival of neurons following injury in both the central and peripheral nervous systems	Namikawa et al., 2000; Guo et al., 2016; Liu et al., 2021; Wang et al., 2020; Akram et al., 2022; Hausott et al., 2022
ATF3	Activating Transcription factor 3	A member of the CREB protein family of transcription factor. Induction of this transcription factors occurs during stress in a variety of tissues, and is known to be upregulated in regenerating neurons. ATF-3 has been documented to help promote regeneration in peripheral nerves.	Nakagomi et al., 2003; Jiang et al., 2004; Seijffers et al., 2006; Seijffers et al., 2007; Akram et al., 2022
BDNF	Brain-Derived Neurotrophic Factor	Neurotrophic factor that is involved in the survival and differentiation of neurons. It has been shown to promote the survival and regeneration of neurons in the central and peripheral nervous systems following injury	Martins et al., 2017; Lucini et al., 2018; Duraikannu et al., 2019; McGregor & English, 2018; Plunet et al., 2002
CHL1	Close Homolog of L1	CHL1 has been implicated in the regulation of cell migration, neurite outgrowth, and synaptic plasticity. CHL1 has also been shown to be upregulated in response to neuronal injury, but studies are conflicting on whether or not CHLI 1 is beneficial or inhibitory to axonal regeneration.	Chaisuksunt et al., 2000; Zhang et al., 2000; Guseva et al., 2018
c-JUN	Transcription factor Jun	Transcription factor that is upregulated following axonal injury. It has been shown to promote axonal regeneration in the CNS	Broude et al., 1997; Zhou et al., 2004; Lee et al., 2022
CREB	cAMP Response Element-Binding Protein	Transcription factor that is involved in the regulation of gene expression. It has been shown to play a critical role in promoting the survival and regeneration of neurons following injury in both the central and peripheral nervous systems	Gao et al., 2004; Teng & Tang, 2006; Tang, 2020; Akram et al., 2022
GAP43	Growth-Associated Protein 43	Neural-specific protein that is highly expressed during axonal regeneration. It promotes axonal growth and guides axons to their targets	Mason et al., 2002; Grasselli & Strata, 2013; Chung et al., 2020
GDNF	Glial cell line-Derived Neurotrophic Factor	Neurotrophic factor that is involved in the survival and differentiation of neurons. It has been shown to promote the survival and regeneration of dopaminergic neurons in the central nervous system	Zhang et al., 2009; Duraikannu et al., 2019; Eggers et al., 2019; Ma et al., 2023
HSP27	Heat Shock Protein 27	Protein upregulated in neurons following traumatic brain injury and to promote neuronal survival	Benn et al., 2002; Hirata et al., 2003; Nakagomi et al., 2003; Hebb et al., 2006; Asthana et al., 2021
HSP70	Heat Shock Protein 70	Protein upregulated in the spinal cord after injury and plays a crucial role in promoting neuronal survival and regeneration	Newfry & Jones, 1998; Nagashima et al., 2011
HSP90	Heat Shock Protein 90	Upregulated in neurons after spinal cord injury and plays a critical role in promoting axon regeneration	Karney-Grobe et al., 2018
IGF-1	Insulin-like Growth Factor 1	Growth factor that is involved in the regulation of cell growth and survival. It has been shown to promote the survival and regeneration of neurons in the central and peripheral nervous systems following injury	Houle et al., 1996; Schmitt et al., 2006; Raivich & Makwana, 2007; Dupraz et al., 2013; Dyer et al., 2016; Akram et al., 2022
KLF7	Krüppel-like Factor 7	Transcription factor that regulates a variety of cellular processes. It has been shown to promote axonal regeneration in the CNS.	Moore et al., 2009; Blackmore et al., 2012; Li et al., 2017; Wang et al., 2017; Liu et al., 2021
LINGO	Leucine-rich repeat and Ig domain-containing Nogo receptor-interacting protein 1	Transmembrane protein that is expressed in both neurons and oligodendrocytes, and it plays a role in regulating axon growth and myelination in the CNS. Inhibition of LINGO-1 has been proposed as a therapeutic strategy for promoting axon regeneration and functional recovery after CNS injury	Rudick et al., 2008; Lv et al., 2010; de Wit et al., 2011; Mi et al., 2013; Quan et al., 2020
NGF	Nerve Growth Factor	Neurotrophic factor that is involved in the survival and differentiation of neurons. It has been shown to promote the survival and regeneration of sensory neurons in the peripheral nervous system	Lindsay, 1988; Fernandez et al., 1990; Jones et al., 2003; Duraikannu et al., 2019
NgR	Nogo Receptor	Receptor that inhibits axonal regeneration in the CNS by binding to myelin-associated inhibitors. Blockade of NgR has been shown to promote axonal regeneration	GrandPré et al., 2002; Hunt et al., 2002; Mi 2008, Yu et al., 2008; Zhang et al., 2008
PKA	Protein Kinase A	Serine/threonine kinase that regulates a variety of cellular processes. Activation of PKA has been shown to promote axonal regeneration	Qiu et al., 2002; Teng & Tang, 2006; Hao et al., 2016; Akram et al., 2022
PTEN	Phosphatase and Tensin homolog	Phosphatase that is involved in the regulation of cell growth and survival. It has been shown to play a critical role in regulating survival and regeneration of neurons following injury in both the central and peripheral nervous systems	Liu et al., 2010; Park et al., 2010; Ohtake et al., 2015; Shabanzadeh et al., 2019; Liu et al., 2021
ROCK	Rho-associated Protein Kinase	Downstream effector of the Rho GTPase signaling pathway that regulates actin cytoskeleton dynamics. Inhibition of ROCK has been shown to promote axonal regeneration	Kubo et al., 2007; Lingor et al., 2007; Tan et al., 2011; Fujita & Yamashita, 2014; Liu et al., 2015; Joshi et al., 2019; Akram et al., 2022; Zheng & Tuszynski, 2023
SCG10	Superior Cervical Ganglion 10	Microtubule-associated protein that is expressed in developing and regenerating axons. It has been shown to promote axonal growth and regeneration in the central and peripheral nervous systems.	Mason et al., 2002; Mori & Morii, 2002; Shin et al., 2014; Li et al., 2023; Li et al., 2024
STAT3	Signal transducer and activator of transcription 3)	is considered a regeneration-associated gene (RAG) that is involved in the process of axon growth. Transcription factor that is activated by a variety of cytokines and growth factors, and plays a role in regulating cell growth, survival, and differentiation. After injury, STAT3 has been shown to be upregulated in neurons and glial cells in the injured CNS, and is involved in the activation of various signaling pathways that promote axon regeneration	Teng & Tang, 2006; Pellegrino & Habecker, 2013; Mehta et al., 2016; Liu et al., 2021; Zheng & Tuszynski, 2023
VEGF	Vascular Endothelial growth factor	Growth factor that is involved in the regulation of blood vessel formation. It has been shown to promote the survival and regeneration of neurons in the central and peripheral nervous systems following injury	Hobson et al., 2000; Mohammadi et al., 2013; Guaiquil et al., 2014
α9β1, α6β1, αvβ3, and αvβ5	Integrin’s	Cell adhesion molecules that are involved in axonal regeneration post injury. These specific integrin proteins have been documented to be expressed by neurons and glia in the injured nervous system and have been shown to play a critical role in axonal regeneration.	Cheah & Andrews, 2018; Nieuwenhuis et al., 2018; Akram et al., 2022

The study of regeneration-associated genes has significantly enhanced our understanding of the intracellular mechanisms involved in neuronal regeneration. More recently, research has focused on the epigenetic regulation of these RAGs and how these mechanisms contribute to the overall post-injury response. Epigenetic modifications play a pivotal role in modulating the expression of RAGs (reviewed in detail by Shin & Cho, 2017; Wahane et al., 2019; Gupta et al., 2023). Collectively, these reviews summarize the complex interplay between epigenetic mechanisms and neural injury responses, particularly in the context of axonal regeneration and glial activation.

Epigenetic modifications, such as DNA methylation, histone modifications (including acetylation, methylation, phosphorylation, and ubiquitination), and non-coding RNAs (like microRNAs and long non-coding RNAs), precisely control gene expression patterns crucial for neuronal responses to injury. Further, epigenetic regulation has been shown to both promote and inhibit axonal regeneration, depending on the specific context and timing of these modifications. For instance, DNA methylation patterns in the promoters of growth-promoting genes, such as those encoding neurotrophic factors or guidance cues, can influence axonal regeneration. Similarly, changes in histone acetylation or methylation at specific lysine residues modulate chromatin accessibility and the recruitment of transcriptional machinery to genes involved in axonal growth and guidance (Shin & Cho, 2017; Wahane et al., 2019; Gupta et al., 2023).

Non-coding RNAs, such as microRNAs and long non-coding RNAs, also exert control over gene expression patterns that orchestrate cellular responses to neural injury. They achieve this by interacting with chromatin-modifying enzymes or directly targeting mRNAs encoding key regulators. For example, microRNAs like miR-9 and miR-21 modulate the post-transcriptional stability and translation of RAG mRNAs, fine-tuning the regenerative response (Wahane et al., 2019; Zhang et al., 2020).

With new insights into the epigenetic regulation of RAGs, new avenues for therapeutic strategies to treat SCI have emerged. These strategies aim to manipulate epigenetic mechanisms to enhance axonal regeneration and reduce inhibitory glial responses. Approaches include using small molecule inhibitors or activators to target epigenetic enzymes, gene therapy to modulate the expression of key epigenetic regulators, and environmental interventions to influence epigenetic modifications (Shin & Cho, 2017; Wahane et al., 2019; Gupta et al., 2023).

### Distance Between the Cell Body & Location of Axotomy

The distance between the axotomy site and the cell body profoundly impacts the ability of neurons to regenerate. Many neuronal types have the capacity to regenerate their axons following injury, but this is influenced by the proximity of the axotomy site to the neuronal cell body, among other factors. A neuron will mount the strongest regenerative response if the site of axotomy is close to the cell body, as indicated by increased axonal growth (David & Aguayo, 1981; Benfey & Aguayo, 1982; Fernandes et al., 1999; Mason et al., 2003).

The expression of RAGs post-injury is often used as an indication that axons are starting to regenerate. To test the effect of lesion distance on RAG expression, CST neurons were axotomized both intracortically and spinally, near and far from the neuronal cell body. There was an upregulation in genes classically associated with regeneration (*Atf3, Gap43, Chl1, Scg10*) in the CST neurons axotomized intracortically near the neuronal cell body, which were not upregulated in CST neurons axotomized spinally (Mason et al., 2003). A similar cellular response was observed in RST neurons (originating in the brainstem) that were subjected to either cervical or thoracic axotomy (Fernandes et al., 1999). Rubrospinal neurons subjected to cervical axotomy upregulated the expression of *Gap43* and various tubulin proteins; this was not evident after thoracic axotomy. The regenerative capacity of retinal ganglion cells (RGCs) was actually mapped out as a function of distance from the lesion (You et al., 2000). Specifically, the authors found that the number of RGCs priming for regeneration significantly decreased as the distance of the lesion site from the retinal ganglion cell soma increased. Similar findings were observed with ReST neurons in lampreys, in a spinal transection model where regeneration was assessed by tracing axonal growth and measuring nerve conduction (Benes et al., 2017). ReST neurons axotomized close to the cell soma showed robust regeneration compared to those axotomized distal to the cell soma. Further, certain populations of PS neurons in the spinal cord show a differential upregulation of RAGs post SCI, depending on the lesion location (Swieck et al., 2019).

It is apparent that axotomy sites proximal to the cell body stimulate more successful axonal regrowth compared to lesions further from the soma. However, there is a critical nuance to this concept: certain neuron populations are especially vulnerable to death when axotomy occurs too close to their cell bodies (reviewed by Rodemer & Selzer, 2019). Specifically, reticulospinal (RST), corticospinal (CST), and dorsal root ganglion (DRG) neurons are particularly prone to retrograde degeneration and cell death adjacent to a lesion. The primary mechanism underlying this susceptibility involves the failure to effectively reseal the axonal membrane when injury occurs near the cell body, leading to persistent calcium influx and activation of apoptotic pathways.

This presents a paradox: while an axotomy in close proximity to the cell body promotes a strong regenerative response, it also increases the risk of neuronal death if it is too close, and the axonal damage extends to the cell body. This suggests that the optimal distance of an axotomy site from the soma aligns with a “Goldilocks principle,” where the lesion must be close enough to elicit a robust intrinsic regenerative response but not so close that it triggers atrophy or death. Neurons too close to the lesion site will be lost, and with larger injuries, there will be significant neuronal loss. This is an important caveat in the development of therapeutic strategies aimed at enhancing neuronal survival and regeneration following spinal cord injuries.

### The Local Inflammatory Response

A rapid inflammatory response, with activation and infiltration of several populations of immune cells, is essential for stimulating robust regenerative sprouting following injury in both the central and peripheral nervous systems (Richardson et al., 1984; Lu & Richardson, 1991; Leon et al., 2000; Fischer et al., 2001; Hossain-Ibrahim et al., 2006; Kigerl et al., 2009; Freyermuth-Trujillo et al., 2022). Early inflammation helps facilitate axonal regeneration by clearing debris, releasing growth factors, and affecting neighboring neurons (Bollaerts et al., 2017). It has been shown to stimulate the regenerative response, including RAG expression in many cell types, including rational ganglion cells (Benowitz & Popovich, 2011; Wong & Benowitz, 2022). The early response involves an influx of immune molecules, some of which will promote axonal regeneration. The vascular macrophage population contains several subclasses, including the M1 and M2 lineages, one of which is considered neurotoxic (M1) and the other beneficial (M2) to axonal regrowth (Kigerl et al., 2009). M2 macrophages appear in the lesion site within the first week, but do not linger; M1 macrophages persist for weeks post-injury (Figure 4). Further, a recently identified subset of granulocytes resembling immature neutrophils, significantly promotes CNS axon regeneration through the secretion of growth factors (Sas et al., 2020).

However, chronic inflammation persists after trauma because of long term degeneration of axons post injury, especially caudal to the lesion. Prolonged inflammation leads to slower Wallerian degeneration, inadequate clearance of tissue debris, and the accumulation of pro-inflammatory cytokines and chemokines, which further inhibits axonal growth and promotes degeneration (Amor et al., 2010). As a result, the chronic inflammatory environment perpetuates the formation and remodeling of the glial scar around the lesion (Fitch & Silver, 2008; Freyermuth-Trujillo et al., 2022). Collectively, this environment promotes continued axonal degeneration, rather than regrowth (Wong & Benowitz, 2022). The dynamics of inflammation plays a critical, but complex role in the regenerative process over time. An important objective in neuroregenerative research requires a comprehensive understanding of the immune response and how it can be modulated to optimize recovery outcomes.

## Neuronal Diversity: Differential Responses to Injury and Implications for Repair

### Corticospinal Neurons

Corticospinal neurons, also known as pyramidal neurons, form the corticospinal tract (CST), a major descending motor pathway originating from the pyramidal neurons in the motor cortex. These neurons have axons that project to alpha motor neurons in the lateral aspect of the spinal cord (Haines, 2012). In humans, the CST is considered the most critical pathway in the control of voluntary movement, leading to significant research focus on repairing the CST after spinal cord injury (SCI).

Recent findings have identified several subtypes of corticospinal neurons, challenging the traditional view of these neurons as a homogeneous population. Segment-specific corticospinal neurons are characterized by distinct projection patterns, with specific spinal cord segments exerting specialized control over movements and muscles (Sahni et al., 2021; Song et al., 2023; Winter et al., 2023). Among these subtypes, Betz cells, located in the primary motor cortex (in the frontal lobe), represent the largest and most well-known subtype. Betz cells project long axons through the brainstem into the spinal cord, where they synapse with alpha motor neurons controlling muscles in the upper and lower extremities (Nolte, 2002; Purves et al., 2008; Strick et al., 2021). However, Betz cells comprise only around 10% of the total population of pyramidal cells in layer Vb (Rivara et al., 2003). Moreover, there is significant variance in axonal diameter among neurons composing the CST (Leenen et al., 1985; Firmin et al., 2014), influencing the speed of action potential conduction and resulting in the categorization into slow and fast CST neurons (Kraskov et al., 2019; Lemon, 2021). Interestingly, while the CST have largely been thought of as an efferent pathway, a subset of corticospinal neurons (CSNs) from the S1/S2 somatosensory cortex directly innervates the spinal dorsal horn, and transecting the CST selectively impairs responses to light touch without affecting noxious stimuli, attenuating tactile allodynia in neuropathic pain (Liu et al., 2018).

Fast corticospinal neurons, though extensively studied due to their role in movement, surprisingly constitute only a small portion of the CST fibers. The majority of CST fibers consist of smaller caliber, slower fibers, which have received less attention due to challenges in their electrophysiological detection (Kraskov et al., 2019; Lemon, 2021). Understanding the differences in CST neurons based on axonal size is crucial with respect to their response to injury. Larger, faster corticospinal neurons are particularly sensitive and vulnerable to trauma like SCI, whereas smaller, slower corticospinal neurons are more resilient (Blight, 1991; Quencer et al., 1992).

### Regenerative Response of Corticospinal Neurons

CST neurons exhibit varied responses depending on factors such as the location of axotomy, the extent of injury, and the age at injury. As noted above, RAG expression is considered an important indicator of the regenerative capacity of neurons; in CST, it is influenced significantly by the proximity of the cell body to the lesion (Mason et al., 2003). Moreover, the presence of an inflammatory agent is sufficient to initiate RAG expression. CST axons of adult rats were cut at the C_3_/C_4_ level, and gene expression in the cell soma was examined following the application of the inflammatory agent lipopolysaccharide (LPS) to the pial surface of the cortex. In situ hybridization and immunohistochemical analysis revealed that CST neurons treated with LPS up-regulated many classic RAGs, including *c-Jun, Atf3, Gap43*, and *Scg10*. However, these genes were not expressed in spinally axotomized CST neurons without LPS treatment. Furthermore, if CST neurons were not axotomized but received LPS treatment, they again showed increased expression of *c-Jun*, *Atf3*, *Scg10*, and *Gap43* (Hossain-Ibrahim et al., 2006). This up-regulation was not observed in the contralateral hemisphere not receiving the LPS. These observations suggest that the local immune response plays a significant role in triggering the initiation of neuronal regeneration.

With a spinal injury RAG upregulation in CST neurons is low or absent, limiting their regenerative potential (Fink et al., 2017). Those that are expressed include transcription factors such as STAT3 and c-Jun, which are involved in activating regeneration-associated gene programs. Interestingly, manipulating the levels of these transcription factors can boost CST axon growth by enhancing the intrinsic growth capacity. The study also highlights the role of mTOR signaling—a pathway that is crucial for cell growth and metabolism—in promoting axon regeneration. Inhibiting PTEN (an mTOR inhibitor) or other suppressors of axon growth has been shown to promote axon regeneration in preclinical models, offering a potential therapeutic strategy to stimulate CST regeneration after CNS injury (Fink et al., 2017). Additionally CST axons have the ability to undergo post injury remodeling with the use of rehabilitative training, which enhance CST axonal sprouting, This is achieved via increased BDNF production which, influences DNA methylation, and produces an increased responsiveness to NTs (Okabe et al., 2017).

More recently, the regenerative potential of CST neurons was demonstrated through use of neural progenitor cell grafts. CST axons elongated through the graft and integrated into the host tissue (Poplawski & Tuszynski, 2020). Interestingly, when the gene expression in CST neurons was examined post injury, there was a noticeable upregulation of the “injury signal”. As the CST axons grew into the neural progenitor cell grafts, the gene expression was more characteristic of a developmental program, rather than regenerative. This suggests that extrinsic signals might drive the regenerative response in CST neurons rather than inherent intrinsic signals. It is unknown whether potential biomolecules or extrinsic signals are being produced in these NPC grafts. It is clear additional studies are needed to characterize how injured CST neurons intrinsically respond to neurotrophic factors, and other pro-regenerative environmental cues/factors. This should include an examination of how each CST subpopulation responds to injury, in terms of RAG expression and axonal growth, to truly assess and maximize the regenerative capabilities of CST.

### Rubrospinal Neurons

Rubrospinal neurons are a group of motor neurons that originate from the red nucleus of the midbrain and extend into the spinal cord. Two main subgroups of neurons emerge from distinct regions of the red nucleus, as detailed by Basile et al. (2021). In primates, the red nucleus comprises the caudal magnocellular region and the rostral parvocellular region (Basile et al., 2021). The magnocellular region contains large, sparse neurons, while the parvocellular region houses small and medium-sized neurons (Basile et al., 2021). Studies indicate that, in primates, neurons from the magnocellular region form the rubrospinal tract, while neurons from the parvocellular region project axons to the ipsilateral inferior olive (Basile et al., 2021).

These neurons are crucial for limb movement and posture control, particularly in non-primate mammals. In humans, the rubrospinal tract’s significance for voluntary movements is relatively minor compared to parallel systems from the cortex and brainstem. However, it coordinates motor function with the CST, especially in upper limb movement coordination. Additionally, the rubrospinal tract is involved in modulating sensory information, suggesting a role in pain and sensory processes (ten Donkelaar, 1988; Miller et al., 1993; Onodera & Hicks, 2009).

### Regenerative Response of Rubrospinal Neurons

There is evidence of post-injury upregulation of certain RAGs in rubrospinal neurons, potentially contributing to their regenerative ability (Fernandes et al., 1999). Examination of rubrospinal neurons following cervical or thoracic axotomy revealed upregulation of Gap43 and various tubulin proteins after cervical injury, which was not observed after thoracic axotomy. Like other neurons, the distance to the lesion strongly influences the intrinsic regenerative capacities of the rubrospinal pathways. Furthermore, treatment with BDNF at the RST cell body upregulates the expression of RAGs, due to the location of TrkB receptors on these neurons. In RST neurons, the TrkB surface receptors are present on the neuronal cell body after injury, but not on the axon. This explains why BDNF treatment at the RST cell body is successful in promoting neuronal survival and axonal sprouting, while treatment at the damaged reactive endings of the axon is not successful (Kwon et al., 2002; Kwon et al., 2004).

The RST may have a unique and important role in functional recovery, serving as an alternate path for nerve impulse transmission. Following injury, new axonal sprouts from both injured and healthy CST neurons establish connections with the rubrospinal tract, creating new neuronal circuits. Studies in mice and primates with CST lesions demonstrated functional recovery, which diminished upon subsequent rubrospinal tract lesions (Fink & Cafferty, 2016; Raineteau & Schwab, 2001). Notably, reorganization of the rubrospinal tract occurs after CST injury, such as increased projections from the red nucleus to basilar pontine nuclei and the raphe nucleus in rodents (Fink & Cafferty, 2016). Primate models show post-injury red nucleus reorganization that resembles CST functionally, with distributed control over flexor and extensor muscles (Raineteau & Schwab, 2001).

### Vestibulospinal Neurons

Vestibulospinal neurons are a group of motor neurons located in the brainstem that play an important role in the control of posture and balance. These neurons receive input from the vestibular system, which is responsible for detecting changes in head position and movement, and use this information to adjust muscle activity in the limbs and trunk for stability. There are two main subtypes of vestibulospinal neurons: medial and lateral.

Medial vestibulospinal neurons originate from the medial vestibular nucleus and project bilaterally to the spinal cord. They primarily control the extensor muscles of the limbs and trunk, which are crucial for maintaining an upright posture and balance (Kandel et al., 2000; Haines, 2012; Mtui et al., 2016). Lateral vestibulospinal neurons originate from the lateral vestibular nucleus and project ipsilaterally (on the same side of the body) to the spinal cord. They regulate both extensor and flexor muscles, which are vital for stabilizing the body during rapid movements like running and jumping (Kandel et al., 2000; Haines, 2012; Mtui et al., 2016). Dysfunction of these neurons can cause balance and posture issues, evident in conditions such as Parkinson’s disease and cerebellar ataxia (Kandel et al., 2000; Haines, 2012; Mtui et al., 2016).

### Regenerative Response of Vestibulospinal Neurons

Regarding their response to injury, this class of neurons has remained largely understudied. Transection experiments in rats have examined the regenerative potential of lateral vestibulospinal tract neurons. Anatomical tracing and functional recovery studies have demonstrated their ability to regenerate and restore connections (Ito et al., 1998). This study appears to be one of the first to specifically demonstrate the regenerative abilities of vestibulospinal neurons. Subsequent work indicated that vestibulospinal neurons can regrow into peripheral grafts and respond to treatments with the bacterial enzyme chondroitinase ABC (cABC) and various neurotrophic growth factors (Jin et al., 2002; Côté et al., 2011). Currently, no studies have specifically examined the intrinsic genetic response of vestibulospinal neurons to axotomy. Considering their role in mobility, posture, and balance, the regeneration of this pathway after trauma or disease will likely be necessary for complete functional recovery. Therefore, additional studies examining the intrinsic response of vestibulospinal neurons to injury are needed.

### Reticulospinal Neurons

Reticulospinal neurons are a group of neurons located in the reticular formation of the brainstem that project to the spinal cord. They are involved in the regulation of a variety of motor functions, including posture, balance, and locomotion. There are two main groups of reticulospinal neurons: the pontine reticulospinal tract (PReST) and the medullary reticulospinal tract (MReST). The PReST originates from the pontine reticular formation and primarily facilitates movement, while the MReST originates from the medullary reticular formation and is involved in both the facilitation and inhibition of movement. Reticulospinal neurons also play a role in the modulation of sensory inputs to the spinal cord and are involved in the generation of reflexes (Brownstone & Chopek, 2018; Perreault & Giorgi, 2019).

### Regenerative Response of Reticulospinal Neurons

Reticulospinal neurons have demonstrated the ability to undergo axonal regeneration following SCI. Many studies highlighting the regenerative capacity of reticulospinal neurons have been conducted in the Sea Lamprey (*Petromyzon marinus*), which possesses a set of large reticulospinal neurons. In this model, ReST neurons will regenerate following axotomy (Jones & Morgan, 2023). In mammalian studies, ReST neurons can show various degrees of axonal regeneration post injury. ReST axons will grow into a progenitor cell-seeded nerve graft placed in a spinal lesion post-injury. Significant axonal ingrowth was observed in the graft, and identification of regenerating neurons was determined via retrograde tracing and antibody staining techniques. Reticulospinal neurons were among the populations that extended new axons into the graft (Jin et al., 2018).

While the intrinsic properties of ReST neurons have yet to be fully characterized, ReST neurons have demonstrated an ability to interact with CST neurons following stroke, SCI, or other injuries. This interaction allows motor commands from the CST to be rerouted through ReST neurons, forming cortico-reticulo-spinal circuits that enable varying degrees of functional recovery in rodent and non-human primate injury models, as well as in some human studies (Zaaimi et al., 2012; Baker & Perez, 2017; Asboth et al., 2018). Interestingly, in a detailed study examining the neuropathology of human spinal cord injury, reticulospinal pathways appeared to be more prone to sparing following injury (Kakulas, 1999). Few, if any, studies have been conducted to characterize the intrinsic regenerative capacity of reticulospinal neurons compared to other neuronal populations. However, given their ability to serve as ‘bypass circuits’ for motor commands from the CST, further studies examining their intrinsic regenerative potential are warranted.

### Dorsal Root Ganglion Neurons

Dorsal root ganglion (DRG) neurons are crucial for transmitting sensory information from the periphery to the central nervous system (CNS). These neurons have a unique pseudounipolar morphology, allowing them to receive information from peripheral sensory receptors and transmit it into the CNS via their projections into the spinal cord (Kandel et al., 2000). *As with other neuronal populations, DRG neurons can also be classified into various subtypes* based on their morphology, electrophysiology, and molecular expression patterns.

Non-peptidergic C-fiber neurons (NP-C) are a subgroup of DRG neurons that express the ion channel Transient Receptor Potential Cation Channel Subfamily V Member 1 (TRPV1) but do not express the neuropeptides calcitonin gene-related peptide (CGRP) or substance P. These neurons are involved in detecting noxious heat and are implicated in chronic pain pathogenesis. NP-C neurons also express the receptor tyrosine kinase RET and the GDNF family receptor alpha 3 (GFRα3), which are essential for their survival and differentiation (Saeed & Ribeiro-da-Silva, 2012; Guo et al., 2022).

Peptidergic C-fiber neurons (PEP-C) are another subgroup of DRG neurons that express both TRPV1 and neuropeptides, including CGRP and substance P. These neurons detect various noxious stimuli and contribute to pain perception and inflammation. PEP-C neurons express the receptor tyrosine kinase RET and the GDNF family receptor alpha 1 (GFRα1), crucial for their development and survival (Pezet & McMahon, 2006). Together, these fiber groups represent about 70% of all primary afferent fibers (Nagy & Hunt, 1983).

Large myelinated A-fiber neurons are involved in detecting proprioceptive and mechanosensory stimuli, such as touch and vibration. They express the mechanosensitive ion channel Piezo2 and the mechanoreceptor-specific transcription factor MafA, and have specialized structures like Meissner’s corpuscles and Pacinian corpuscles (Arcourt et al., 2017).

Small myelinated A-fiber neurons detect mechanical and thermal stimuli and are also nociceptive. They express ion channels and receptors such as TRPV1, TRPM8, and Nav1.8, playing a critical role in pain conditions and pain management (Abrahamsen et al., 2008). The variable axonal sizes in the DRG are related to the expression of p75NTR, which can group neurons into subpopulations based on axon diameter (Murray & Cheema, 2003).

### Regenerative Response of Dorsal Root Ganglion Neurons

DRG neurons have two projections: one in the peripheral nervous system (PNS) and one in the CNS. Their response to axotomy depends on which projection is injured. Axon regeneration is more robust in the PNS compared to the CNS due to the more permissive environment in the PNS. After an axon injury in the PNS, Schwann cells near the injury site undergo dedifferentiation and proliferation, creating a growth-promoting environment for regenerating axons. These Schwann cells produce extracellular matrix and growth-promoting factors that aid axonal regeneration (Filbin, 2003; Cattin et al., 2015; He & Jin, 2016; Fawcett & Verhaagen, 2018). This triggers the expression of regeneration-associated genes (RAGs) such as *Gap43, Atf3, Galanin, Sox11*, and *c-jun*, which are associated with axonal regeneration (Skene & Willard, 1981; Holmes et al., 2000; Raivich & Makwana, 2007; Jankowski et al., 2006; Seijffers et al., 2007; Liu & Li, 2009). Galanin is notably upregulated at high levels in DRGs following peripheral injury and serves as a marker for regenerating DRGs (Bacon et al., 2007). The p75 receptor is also upregulated in DRG neurons following axotomy and promotes axon growth and regeneration. p75 interacts with other receptors, such as TrkA and SorCS2, to modulate DRG neuron responses to neurotrophins and other growth factors (Hempstead, 2002; Teng et al., 2005). The upregulation of p75 and its interactions with other receptors are crucial for stimulating axon regeneration after injury.

Following peripheral axotomy, DRG neurons also upregulate surface receptors for molecules that promote survival and axonal growth. For example, they increase the expression of the receptor tyrosine kinase RET, the high-affinity receptor for glial cell line-derived neurotrophic factor (GDNF), which is secreted by Schwann cells and aids DRG neuron survival and growth (Tomita et al., 2007). DRG neurons also upregulate the high-affinity receptor for nerve growth factor (NGF), TrkA, and the high-affinity receptor for brain-derived neurotrophic factor (BDNF), TrkB (Molliver et al., 1995). These neurotrophic factors promote the survival and growth of sensory neurons in the PNS. Increased expression of these surface receptors contributes to DRG neuron survival and axonal regeneration after injury.

In contrast, the CNS environment is less permissive for axon regeneration. Axotomy of central projections results in a lower capacity for axonal regeneration, despite the expression of RAGs. CNS injury also leads to the expression of the inhibitory gene *Gsk3β* (Herdegen et al., 1991; Raivich et al., 2004; Seijffers et al., 2007; Liu et al., 2010; Bareyre et al., 2011; Diekmann & Fischer, 2015; Leibinger et al., 2017). *GSK-3β* negatively regulates axonal regeneration by inhibiting the Wnt/β-catenin signaling pathway, and its inhibition can enhance CNS axonal regeneration (Leibinger et al., 2017; Diekmann & Fischer, 2015).

DRG neurons also respond to inflammation after injury. For instance, injecting the bacterium *Corynebacterium parvum* into the DRG after a crush injury significantly increased DRG axonal outgrowth in the dorsal columns compared to controls (Lu & Richardson, 1991). Another inflammatory agent, zymosan, applied to DRG neurons, has improved dorsal root regeneration following axotomy (Steinmetz et al., 2005).

These studies collectively demonstrate that DRG neurons are a diverse population with regenerative capacity dependent on the projection injured. DRG neurons in the PNS exhibit a greater capacity for axon regeneration compared to those in the CNS. PNS DRG neurons can regenerate axons over long distances and reinnervate target tissues, while CNS DRG neurons have more limited regeneration capacity, often failing to reach their targets. DRG neurons respond to inflammation post-injury, but significant spinal cord trauma may impede their survival.

### Propriospinal Neurons

Propriospinal neurons (PSNs) are a population of interneurons that interconnect different levels of the spinal cord. Unlike supraspinal neurons (e.g., corticospinal, rubrospinal, reticulospinal, or vestibulospinal neurons) that originate in the brain or brainstem and project axons into the spinal gray matter, PSNs both originate and terminate within the spinal cord (Chung & Coggeshall, 1983). Anatomical studies show that PSN axons comprise approximately one-quarter to one-third of the fiber tracts in the dorsal and dorsolateral funiculi of the rat spinal cord (Chung et al., 1987). PSNs are classified into two major groups: long and short axon PS neurons (Conta & Stelzner, 2004; Conta & Stelzner, 2009). These groups are further divided into subgroups such as long ascending PS neurons, long descending PS neurons, short thoracic PS neurons, short axon PS neurons, thoracic respiratory PS neurons, and upper cervical respiratory PS neurons. Detailed descriptions of these subgroups can be found in Conta & Stelzner, 2009.

Long descending propriospinal tract (LDPT) neurons are located within the intermediate gray matter (laminae VII and VIII), deeper laminae of the dorsal horn (laminae V and VI), and around the central canal (lamina X) of the cervical enlargement (CE) (Figure 1; Conta & Stelzner, 2004; Conta & Stelzner, 2009). After extending their axons caudally for many spinal segments, their main axonal projection terminates in the intermediate gray matter of the lumbosacral enlargement (LSE). Conversely, long ascending propriospinal tract (LAPT) neurons, originating in the rostral segments of the LSE, project rostrally towards the CE. Their main projections terminate in the intermediate gray matter of the CE, connecting both spinal enlargements. LDPT neurons are thought to mediate limb coordination (e.g., Jankowska et al., 1974; Stelzner & Cullen, 1991) and may be involved in the Central Pattern Generator (CPG) for locomotion (Jordan & Schmidt, 2002).

Short thoracic propriospinal (TPS) neurons, as their name suggests, arise from the thoracic spinal cord and can be found in all laminae of the gray matter except lamina IX. These neurons extend their projections from 1 to 5 segments in either the rostral or caudal direction (Conta & Stelzner, 2004; Conta & Stelzner, 2009). Previous studies have shown that TPS neurons are primarily involved in the axial musculature and posture (Anderson, 1963; Vasilenko, 1975).

### Regenerative Response of Propriospinal Neurons

The injury response of propriospinal neurons is variable, depending on the populations and other factors. Gene profiling studies have revealed significant differences between certain populations, depending on the injury location. Utilizing a complete spinal cord transection model at the level of T_9_, TPS neurons exhibited a dynamic and robust up-regulation of genes associated with axonal regeneration (RAGs), neuroprotection, neurotrophic/growth factors, surface receptors, and pro-apoptotic function (Siebert et al., 2010a). Following the T_9_ level axotomy, TPS neurons up-regulated Lifr, a receptor for LIF and a co-receptor for CNTF, while no significant change in expression of any of the Ntrk receptors (1, 2, or 3) was observed post-injury.

This was in stark contrast to the dramatic down-regulation or lack of significant response in genes encoding surface receptors, neurotrophic factors, RAGs, and cell death genes within LDPT neurons (Siebert et al., 2010b). A surprising discovery was the phenotypic differences found between TPS and LDPT neurons. The genes regulating the expression of many potent neurotrophic factor surface receptors (Cntfr, Gfra1, Gfra2, Lifr, Ntrk1, and Ntrk2) and neurotrophic factors (Artn, Ntf5) were expressed at significantly higher levels in LDPT neurons than in TPS neurons. While the reasons for these phenotypic differences are unknown, one hypothesis is that the large difference in axonal length between the two populations of PS neurons is responsible. LDPT axons project over a long distance between the two spinal enlargements, while TPS axons have much shorter projections. More neurotrophic support may be needed to cope with the increased metabolic demands necessitated by the long axons of LDPT neurons.

Furthermore, more detailed studies revealed that the response of the LDPT to injuries at different levels was related to the distance from the lesion (Swieck et al., 2019). LDPT generally exhibit neither a regenerative nor an apoptotic response to injuries that occur very distal to their cell bodies, such as at T_10_ (Swieck et al., 2019). However, when a lesion occurs at the T_2_ level, LDPT neurons display a response similar to that of TPS neurons. This indicates that the lesion distance from the cell bodies can alter the ability to regenerate (Swieck et al., 2019). Overall, neurons produce the greatest regenerative response when the injury occurs close to cell bodies. Local axotomy of both TPS and LDPT populations produced a robust increase in immune and inflammatory gene expression. Apoptotic gene expression was also observed in TPS and LDPT tracts after local, but not distant, axotomy. A critical transcriptional regulator of the regenerative response called Tbp, which stands for TATA-binding protein, is significantly more upregulated in TPS compared to LDPT neurons, which have little expression of Tbp (Swieck et al., 2019). Lastly, growth factor expression increased in TPS post-injury but not in LDPT. One possible reason for the differences between TPS and LDPT, even when they both undergo local axotomy, is that LDPT may have ‘sustaining collaterals,’ or input from other nearby neurons. These collaterals would support the LDPT, likely alleviating cellular signals originating from the injury that would trigger a regenerative response. Another potential explanation is that there are fundamental differences between TPS and LDPT cell populations, as evidenced by gene expression. Overall, these findings suggest that both lesion distance and neuronal population subtype play a role in plasticity and the regenerative responses of this neuronal population.

Propriospinal neurons show a remarkable post-injury plasticity, allowing for the formation of functional bypass circuits after an incomplete SCI. They will connect with CST neurons, forming a pathway around the lesion which can restore motor function (Courtine et al., 2008; Stelzner, 2008). Later studies have demonstrated that PSN will also form bypass circuits through interactions with other populations of neurons like reticulospinal neurons, allowing for varying degrees of motor recovery (Filli et al., 2014; May et al., 2017). The plasticity of propriospinal neurons and their interactions with other neuronal populations offer an alternative therapeutic strategy to achieve functional recovery (reviewed by Filli & Schwab, 2015). Collectively this literature suggests that recovery from spinal cord injury may be possible through the functional reorganization of spinal circuitry, without complete regeneration of the SSNs.

### Serotonergic (5-HT) Neurons

Serotonin (5-hydroxytryptamine, 5-HT) neurons represent a distinct subset within the diverse population of neurotransmitter-producing cells in the central nervous system. These neurons predominantly inhabit the raphe nuclei situated within the brainstem. Anatomical investigations into the localization of 5-HT cell bodies and their corresponding projections have delineated two major subgroups. The first group, situated rostrally, is found in the mesencephalon and the rostral pons, with these neurons extending their axons into the forebrain. In contrast, the second subgroup, located more caudally in the rostral pons and medulla oblongata, sends their axonal projections downward into the brainstem and spinal cord (Charnay & Léger, 2010). Apart from their common phenotype, 5-HT neurons also exhibit varying cellular characteristics encompassing anatomy, morphology, pathway architecture, electrophysiology, and gene expression. Notably, there is differential expression of molecules facilitating the co-transmission of additional neurotransmitters among these neurons. This intricate diversity implies the existence of functionally heterogeneous subtypes within the 5-HT neuron population. However, establishing precise associations between subsets of these neurons and specific functions has presented formidable technical challenges and remains problematic (Okaty et al., 2019). Collectively, serotonergic neurotransmission plays a vital role in regulating sensory, motor, and autonomic functions within the spinal cord. The serotonergic system contributes to the regulation of axonal growth, guidance, and synaptogenesis, thus holding significant promise in the development of therapeutic strategies aimed at enhancing axonal regeneration following neural trauma or disease.

### Regenerative Response of 5-HT Neurons

Axonal sprouting from 5-HT neurons after SCI has been well documented, and is unique in that it is somewhat unaffected by the inhibitory molecules present at the lesion site (Hawthorne et al., 2011). Typically, 5-HT axons will degenerate caudally, particularly in more severe injuries, while above the lesion, significant sprouting and increased 5-HT axon density are often observed. This pattern is consistent in both rats and mice across various injury models (Perrin & Noristani, 2019). Dorsal 5-HT projections are especially prone to degeneration, whereas ventral projections are generally spared. The increase in 5-HT axon density near the lesion site post-injury suggests that these neurons are predisposed to regenerate. This regenerative response is a hallmark of the 5-HT system and is also observed in other central nervous system injuries and neurodegenerative conditions (Perrin & Noristani, 2019). The cellular mechanisms underlying this enhanced sprouting response remain unclear at present.

### Taking Advantage of Neuronal Diversity in Achieving Functional Recovery

The ultimate goal in the study of SCI is to restore motor and sensory function. Scientific research has often focused on promoting the regeneration of damaged neurons and their reinnervation of original targets. However, a distinction must be made between anatomical recovery and functional recovery. Anatomical recovery generally refers to the regrowth of injured axons across the lesioned area. This regrowth is often limited by the inhibitory environment present after injury and the intrinsic growth capacity of the individual neurons. An increase in axon growth, especially following therapeutic treatments, does not necessarily ensure that these axons will reconnect with their original targets and restore motor function. Functional recovery can also be achieved through the reorganization of neural connections, which results from axonal sprouting and synapse formation on new neurons. This neuroplasticity has been observed in experimental models using treatments that promote anatomical recovery and is likely involved in most cases where motor function is partially restored. Currently, there is a significant lack of substantial evidence that recovery after SCI is directly due to regenerated axons from a single neuronal population (Tuszynski & Oswald, 2012; Fink & Cafferty, 2016). Thus, motor recovery is likely achieved through a combination of new axonal regrowth and the establishment of new pathways involving multiple neuronal populations, facilitated by rehabilitative training and reorganization of the motor cortex to accommodate new circuits (Brown & Martinez, 2019).

Propriospinal (PS) neurons are often a key component in forming new circuits. They have demonstrated the ability to create functional neuronal ‘bypass’ circuits around the lesion site, allowing for recovery of function following incomplete spinal injury and interruption of long descending motor tracts (Bareyre et al., 2004; Courtine et al., 2008; Stelzner, 2008). Sprouting from intact supraspinal axons post-injury has been observed in certain neuronal populations, such as the CST, ReST, and RST. Neighboring intact CST neurons can sprout locally and coordinate with other neuronal populations to restore connections to their targets (reviewed by Onifer et al., 2011; Fink & Cafferty, 2016; Brown & Martinez, 2019). These new CST axons often synapse on propriospinal neurons (Courtine et al., 2008; Doperalski et al., 2020), which then synapse on lower motor neurons that innervate muscles. In this way, motor function can be restored by forming alternative neuronal circuits.

The ability of neurons to form synaptic connections with multiple neuronal subpopulations, resulting in the creation of functional bypass circuits, raises an important question for therapeutic strategies: Is it more effective to focus on coaxing a specific neuronal population or class to undergo a complete regenerative response, or to stimulate the formation of new bypass circuits?

### Final Thoughts

It has been 41 years since the studies of David & Aguayo revealed that CNS neurons possess inherent regenerative capabilities when provided with an appropriate growth-promoting environment. Technological advancements now allow us to evaluate the intrinsic responses of distinct neuronal populations and identify unique features of neuronal subsets. Recent transcriptomic analysis of brain tissue has revealed unexpectedly diverse populations of neurons, with individual specializations localized to specific brain regions (Siletti et al., 2023). Neuronal subpopulations exhibit differences in susceptibility to growth factors, environmental influences, and regenerative potential. Currently, there is a lack of information on the intrinsic responses of neuronal subpopulations to axotomy. Single-cell RNA analyses of spinal cord lesions may be valuable in identifying cellular responses, as demonstrated when such analyses were used to identify regeneration-associated genes (RAGs) in ascending spinocerebellar neurons in the lumbar spinal cord post-injury (Matson et al., 2022).

Neurotrophins have been often tested in spinal cord injury models to promote neuronal regeneration and functional recovery (reviewed by Harvey et al., 2015) This includes NGF, BDNF, NT-3, and GDNF, all of which have been tested alone or in combination as SCI treatments. The results are mixed, due to a number of considerations that have not been well defined. Significant concerns include the mode and timing of neurotrophin delivery post-injury, as there is no clear understanding of a good timeline for treatment. Another key consideration that warrants study is the neuronal populations and how they respond to neurotrophins over time after an injury. One potential explanation for the varied functional recovery rates could be the selective expression of neurotrophin receptors on specific neurons. Examination of the *intrinsic* properties of specific neuronal populations have identified a number of attributes, including receptor expression, that may contribute to their ability to survive, regenerate and restore connections after injury.

Is functional recovery the result of axonal regeneration, neuronal plasticity and circuit reorganization, the glial response, or a combination of these processes? These are difficult questions to answer, even with emerging technologies. It is not merely the vast array of neuronal populations but also the effect of the local environment on the behavior of these different subpopulations post-injury that remains unknown. We can no longer view neuronal populations (e.g., CST, RST, VST, ReST, PST) as homogeneous entities but rather as a diverse assemblage of subpopulations. To make significant strides in SCI research, it is crucial that we explore and truly appreciate this breadth of neuronal diversity.

## Abbreviations

CNS: Central Nervous System
BDNF: Brain-derived neurotrophic factor
CPG: Central Pattern Generator
CE: Cervical enlargement
CSPGs: Chondroitin sulfate proteoglycans
cABC: Chondroitinase ABC
CST: Corticospinal tract
DAMPs: Damage-associated proteins
GDNF: Glial cell line-derived neurotrophic factor
GAG: Glycosaminoglycan
LPS: Lipopolysaccharide
LAPT: Long Ascending PS neurons
LDPT: Long descending PS neurons
LSE: Lumbosacral enlargement
NGF: Nerve growth factor
OPCs: Oligodendrocyte progenitor cells
PS: Propriospinal
RAGs: Regeneration-associated genes
ReST: Reticulospinal tract
RGC: Retinal ganglion cells
RST: Rubrospinal tract
5-hydroxytryptamine, 5-HT: Serotonin
TPS: Short thoracic PS neurons
SSNs: Supraspinal neurons
VST: Vestibulospinal tract
